# Clofazimine inhibits innate immunity against *Mycobacterium tuberculosis* by NF-κB

**DOI:** 10.1128/msphere.00254-24

**Published:** 2024-07-24

**Authors:** Xinda Li, Xiaoyi Luo, Bin Wang, Lei Fu, Xi Chen, Yu Lu

**Affiliations:** 1 Department of Pharmacology, Beijing Chest Hospital, Capital Medical University, Beijing, China; 2 Beijing Key Laboratory of Drug Resistance Tuberculosis Research, Beijing Tuberculosis and Thoracic Tumor Research Institute, Beijing, China; Hackensack Meridian Health Center for Discovery and Innovation, Nutley, New Jersey, USA

**Keywords:** *Mycobacterium tuberculosis*, antitubercular drug, post-tuberculous lung disease, immunomodulation

## Abstract

Tuberculosis (TB) remains one of the infectious diseases with high incidence and high mortality. About a quarter of the population has been latently infected with *Mycobacterium tuberculosis*. At present, the available TB treatment strategies have the disadvantages of too long treatment duration and serious adverse reactions. The sustained inflammatory response leads to permanent tissue damage. Unfortunately, the current selection of treatment regimens does not consider the immunomodulatory effects of various drugs. In this study, we preliminarily evaluated the effects of commonly used anti-tuberculosis drugs on innate immunity at the cellular level. The results showed that clofazimine (CFZ) has a significant innate immunosuppressive effect. CFZ significantly inhibited cytokines and type I interferons (IFNα and IFNβ) expression under both lipopolysaccharide stimulation and CFZ-resistant strain infection. In further mechanistic studies, CFZ strongly inhibited the phosphorylation of nuclear factor kappa B (NF-κB) p65 and had no significant effect on the phosphorylation of p38. In conclusion, our study found that CFZ suppresses innate immunity against *Mycobacterium tuberculosis* by NF-κB, which should be considered in future regimen development.

**IMPORTANCE:**

The complete elimination of *Mycobacterium tuberculosis* (Mtb), the etiologic agent of TB, from TB patients is a complicated process that takes a long time. The excessive immune inflammatory response of the host for a long time causes irreversible organic damage to the lungs and liver. Current antibiotic-based treatment options involve multiple complex drug combinations, often targeting different physiological processes of Mtb. Given the high incidence of post-tuberculosis lung disease, we should also consider the immunomodulatory properties of other drugs when selecting drug combinations.

## INTRODUCTION

Tuberculosis (TB), caused by infection with *Mycobacterium tuberculosis*, is an ancient disease (1). Because of its strong infectivity and high fatality rate, it has ranked first among infectious diseases for a long time. According to the World Health Organization, about 1.5 million people die from TB each year, and a quarter of the world’s population has a latent infection with *Mycobacterium tuberculosis*, of which about 5%–10% will develop active TB (2–4). Currently, the treatment for sensitive TB is the simplest, which still needs to be combined with four front-line anti-TB drugs for 6 months, while the treatment of multidrug-resistant TB (MDR-TB) needs to choose more drugs according to different drug resistance characteristics, and the treatment time may be extended to 18–20 months, or even longer (3, 5–10).

After such a long period of antibiotic treatment, the sequelae of TB patients and the impact on the quality of life have gradually aroused people’s attention (11–14). Akkara et al. documented that 85% of patients had respiratory symptoms, such as cough, sputum, or dyspnea, lasting an average of 8.9 months after TB treatment, and 12% had hemoptysis (15). In the study by Singla et al., 87% of 46 patients with MDR-TB had dyspnea after completing 2 years of treatment, the highest incidence of sequelae, and 65% had modified Medical Research Council (mMRC) grade 2 dyspnea (16). In another study, cough (94%) was also the main sequelae, followed by chest pain (63%) and sputum (51%) accompanied by dyspnea and hemoptysis (34% and 28%, respectively)(14, 17).

The uncontrolled long-term overproduction of cytokines is an important factor causing lung injury in patients with tuberculosis (18–20). For example, tumor necrosis factor alpha (TNF-α) can drive necrosis and irreversible lung injury. Many studies have reported that the induction of TNF-α after tuberculosis is associated with clinical deterioration and severe tissue injury (21, 22). Interleukin-1β (IL-1β) and IL-17 can directly recruit neutrophils, affect pulmonary fibrosis, and is positively correlated with disease severity and the size of pulmonary cavities (23–25). TNF induces pathogenic mitochondrial reactive oxygen species (ROS) in tuberculosis. Excessive production of reactive oxygen species can lead to oxidative imbalance in the lung, and further trigger the release of pro-inflammatory mediators, making the inflammatory response continue and intensify, and then lead to tissue damage (26).

In this study, we initially evaluated the effects of commonly used anti-tuberculosis drugs including isoniazid, rifampicin, pyrazinamide, ethambutol, clofazimine, TBI-166, and bedaquiline on innate immunity. Remarkably, clofazimine significantly inhibited the activation of innate immunity. Clofazimine, also reported in a previous review (27), has anti-inflammatory/immunosuppressive properties and can be used in the treatment of discoid lupus erythematosus, pustular psoriasis, Melkersson–Rosenthal syndrome, necrobiosis lipoidica, and granuloma annulare, as well as cutaneous lesions in systemic lupus erythematosus (27, 28). However, the immunomodulatory effects of clofazimine in the treatment of tuberculosis are not well understood.

We evaluated the effect of clofazimine on cytokine production in macrophages by treating macrophages with lipopolysaccharide or infecting macrophages with clofazimine-resistant strains. We observed that clofazimine significantly decreased the cytokines IL-6, TNF-α, IL-1β, and alpha and beta interferon (IFNα and IFNβ). In the CFU experiment, CFZ had no effect on the growth of CFZ-resistant strains in macrophages. Furthermore, we demonstrated that CFZ may play an anti-cytokine role by inhibiting the phosphorylation of nuclear factor kappa B (NF-κB) p65. In conclusion, CFZ plays an important role in the anti-Mtb immune balance of the host.

## RESULTS

### Clofazimine inhibits innate immune signaling pathways

**Fig 1:**
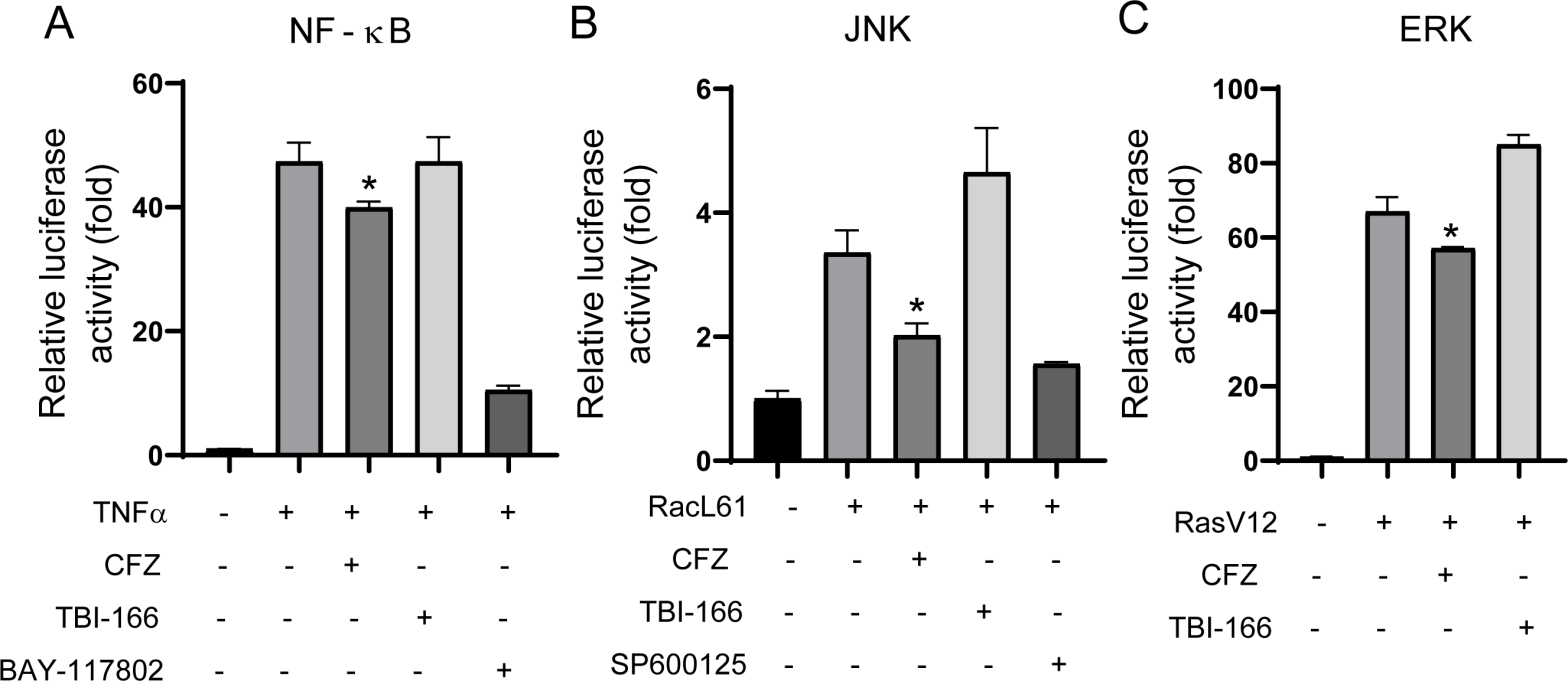
CFZ inhibits the activation of innate immune signaling pathways. (A to C) HEK293T cells were seeded into 24 wells, transfected with NF-κB (A), AP-1 (B), or Elk (C) luciferase reporter plasmid and treated with CFZ (2 µg/mL) or an equal volume of dimethylsulfoxide for 24 h. The Elk was activated by co-expression of constitutively active RasV12. The AP-1 was activated by co-expression of constitutively active RacL61. The NF-κB pathway was stimulated by TNF treatment. BAY-117802, a known NF-κB inhibitor, and SP600125, a known AP-1 inhibitor, were used as positive controls in this study. Then, the cell lysates were harvested for a luciferase assay (*n* = 3; means and SD; *, *P* < 0.05; **, *P* < 0.01; two-sided *t*-test).

To assess the effects of currently commonly used anti-tuberculosis drugs on host innate immune signaling pathways, we used a dual fluorescence reporting system to detect drug effects on NF-κB pathway, JNK pathway, and ERK pathway in HEK293T, including isoniazid (INH), rifampicin (RFP), pyrazinamide (PZA), ethambutol (EMB), clofazimine, TBI-166, and bedaquiline (BDQ). Among them, CFZ inhibits the activation of all three of the above signaling pathways, as shown in Fig. 1A, B, and C. Data for other drugs are shown in Fig. S1.

### CFZ decreases the expression of cytokines and type I interferon

**Fig 2:**
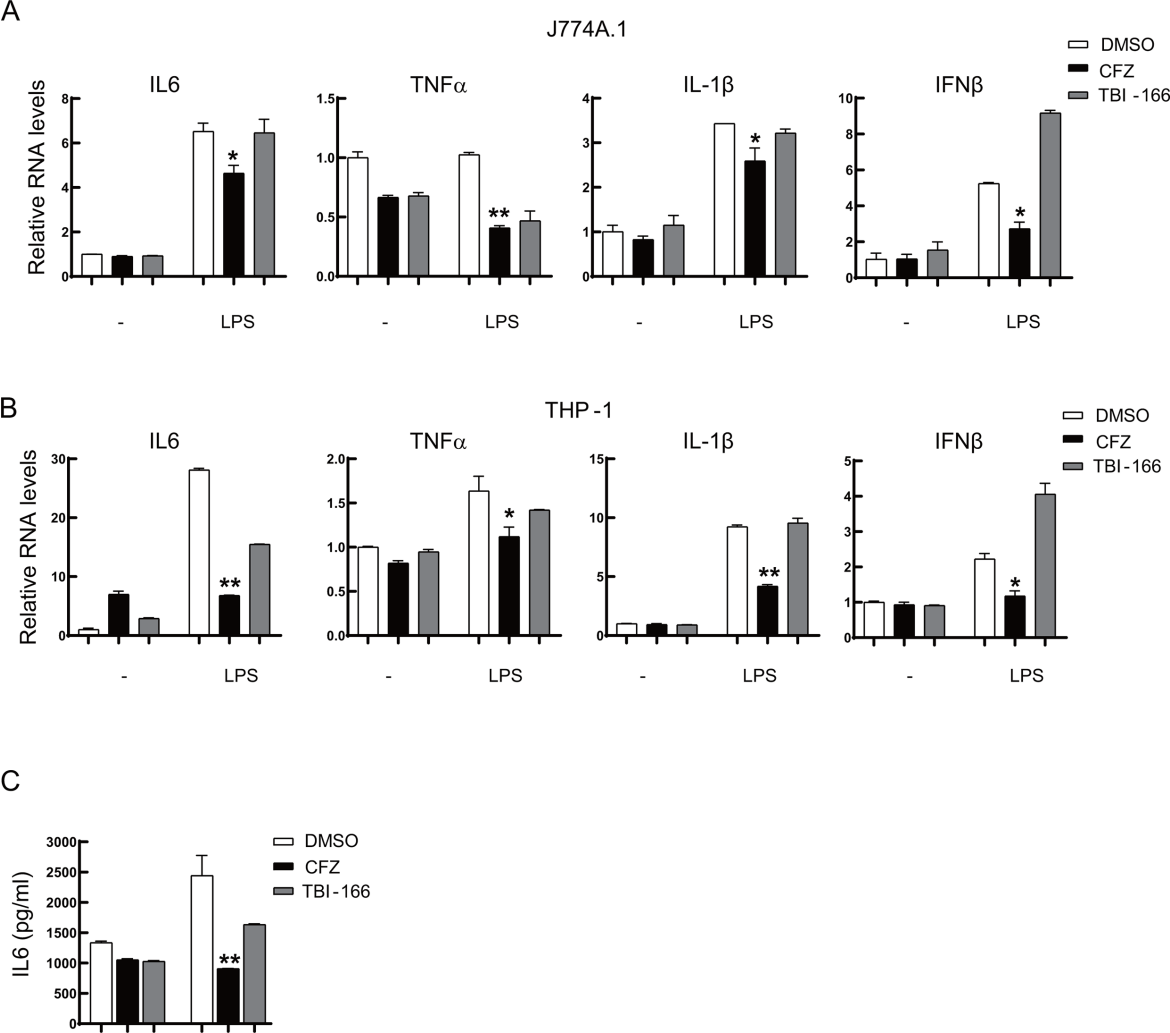
Under LPS stimulation, CFZ inhibits the expression of cytokines and type I interferon. (A to C) J774A.1 macrophages (A) and PMA-induced THP-1 macrophages (B) were stimulated with LPS (100 ng/mL) for 12 h and treated with CFZ (2 µg/mL) or an equal volume of DMSO for 24 h. Then, the total RNA and supernatants were prepared. The mRNA levels of the cytokines and type I interferon were determined by RT-qPCR (**A and B**) (*n* = 3; means and SD; *, *P* < 0.05; **, *P* < 0.01). The supernatants were subjected to ELISA assay for IL-6 (C) (*n* = 3; means and SD; *, *P* < 0.05; **, *P* < 0.01; two-sided *t*-test).

To identify the effect of CFZ on cytokine production, we first treated J774A.1- and PMA-induced THP-1 macrophages with lipopolysaccharide (LPS) to simulate the infection of macrophages by pathogens and treated them with CFZ for 12 h. The cells were then collected, and RNA was extracted, and followed by RT-qPCR to detect IL-6, TNFα, IL-1β, and IFNβ. The supernatants were used in ELISA experiments. The results showed that CFZ and TBI-166, a structural analog of CFZ, inhibited the production of these cytokines and IFNβ (Fig. 2A and B). The expression of IL-6 in the supernatant of CFZ treatment group was significantly lower than that of the dimethylsulfoxide (DMSO) group (Fig. 2C).

**Fig 3:**
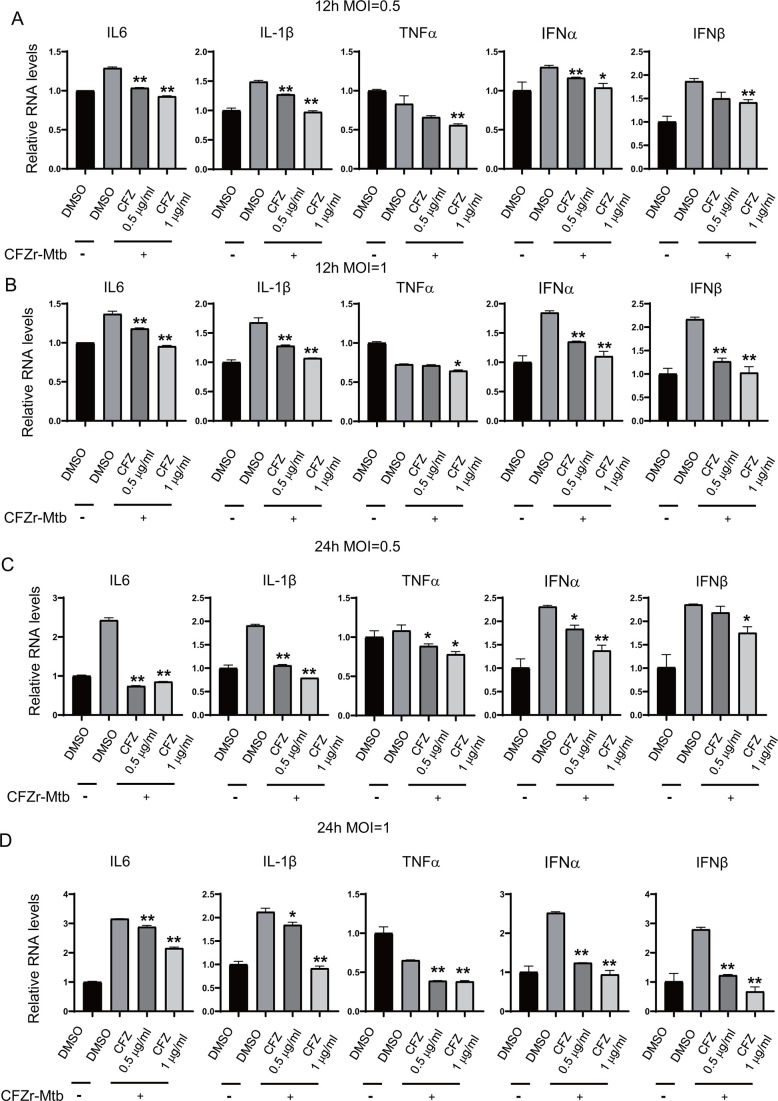
CFZ decreases the expression of cytokines and type I interferon after infection with CFZr-Mtb. (**A, B, C, and D**) J774A.1 macrophages were plated into six wells for 18 h. Then infected with CFZr-Mtb at a multiplicity of infection (MOI) of 0.5 (A) or MOI of 1 (**B**) for 4 h, and treated with CFZ (0.5 µg/mL), CFZ (1 µg/mL), or DMSO for 12 h (**A and B**) or 24 h (**C and D**). Cells were collected for RNA extraction to detect the transcription level of IL-6, IL-1β, TNF-α, IFNα, and IFNβ (*n* = 3; means and SD; *, *P* < 0.05; **, *P* < 0.01; two-sided *t*-test).

**Fig 4:**
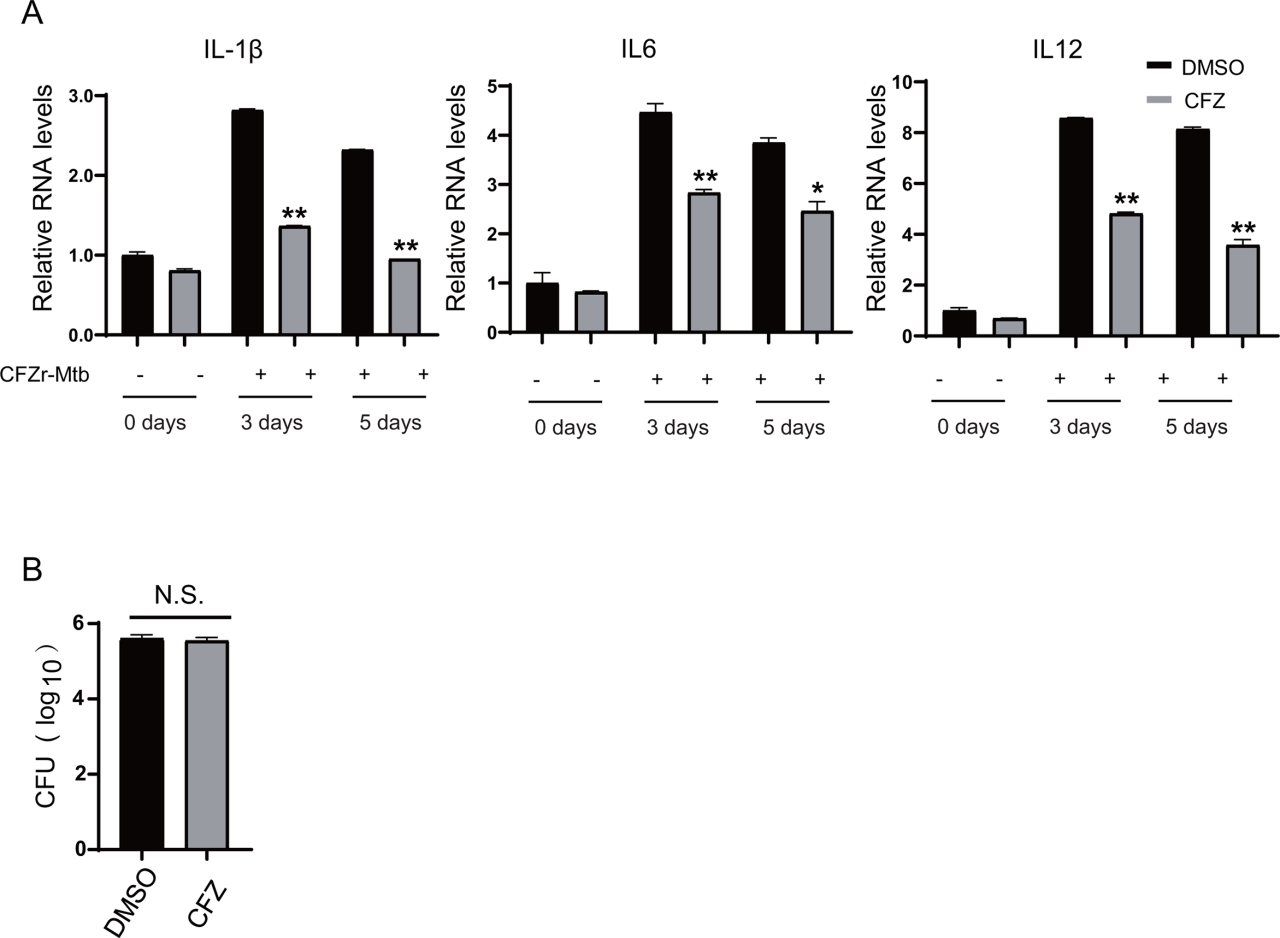
CFZ did not promote the intracellular growth of CFZr-Mtb. (**A**) J774A.1 macrophages were plated into six wells for 18 h. And then infected with CFZr-Mtb at an MOI of 1 for 4 h, and treated with CFZ (1 µg/mL) or DMSO for 3 days or 5 days. Cells were collected for RNA extraction to detect the transcription level of IL-6, IL-1β, TNF-α, IFNα, and IFNβ (*n* = 3; means and SD; *, *P* < 0.05; **, *P* < 0.01; two-sided *t*-test). (**B**) J774A.1 macrophages were infected with CFZr-Mtb at an MOI of 1 for 4 h and treated with CFZ (1 µg/mL) for 24 h. Cells were harvested and intracellular survival of CFZr-Mtb was determined by performing colony forming unit (CFU) assay (*n* = 3; means and SD; *, *P* < 0.05; **, *P* < 0.01; two-sided *t*-test).

We infected J774A.1 macrophages with a clinically isolated CFZ-resistant Mtb strain (CFZr-Mtb) and measured the mRNA levels of type I interferon IFNα and IFNβ and cytokines IL-6, IL-1β, and TNF-α. The minimum inhibitory concentration (MIC) of CFZ against CFZr-Mtb was 3.17 µg/mL. The final concentrations of CFZ we used are 0.5 µg/mL and 1 µg/mL. Consistent with the results of LPS treatment, CFZ significantly inhibited the expression of IL-6, IL-1β, TNF-α, IFNα, and IFNβ (Fig. 3A, B, C, and D). We examined the effect of CFZ treatment on cytokine production for a longer period of time, and the results showed that the inhibitory effect on cytokines persisted after 3 and 5 days of CFZ treatment (Fig. 4A).

### CFZ does not affect the survival of CFZr-Mtb in macrophages

We wanted to know whether the regulation of CFZ on host innate immunity affects the growth of CFZr-Mtb within macrophages. We infected J774A.1 cells with CFZr-Mtb (MOI = 1) for 4 h, then continued treatment with CFZ (1 µg/mL) for 48 h, and counted intracellular Mtb CFU. The data showed that CFU counts were similar in the CFZ treatment group and the DMSO treatment group, with no significant differences (Fig. 4B). The antibacterial effects of IL-6, TNF-α, and IL-1β and the growth-promoting effects of IFNα and IFNβ were neutralized under the treatment of CFZ, and the intracellular survival was not significantly affected.

### RNA deep sequencing reveals that CFZ inhibits the production of cytokines and chemokines

To further verify the inhibition of CFZ on the activation of innate immune signaling pathways, we conducted RNA deep sequencing in J774A.1 macrophage. J774A.1 cells were infected with CFZr-Mtb (MOI = 1) for 4 h and continued to be treated with CFZ (0.5 µg/mL or 1 µg/mL) for 24 h. At the same time, the uninfected group was used as the control group, and all cells were collected for RNA deep sequencing. Each group contained three replicates.

**Fig 5:**
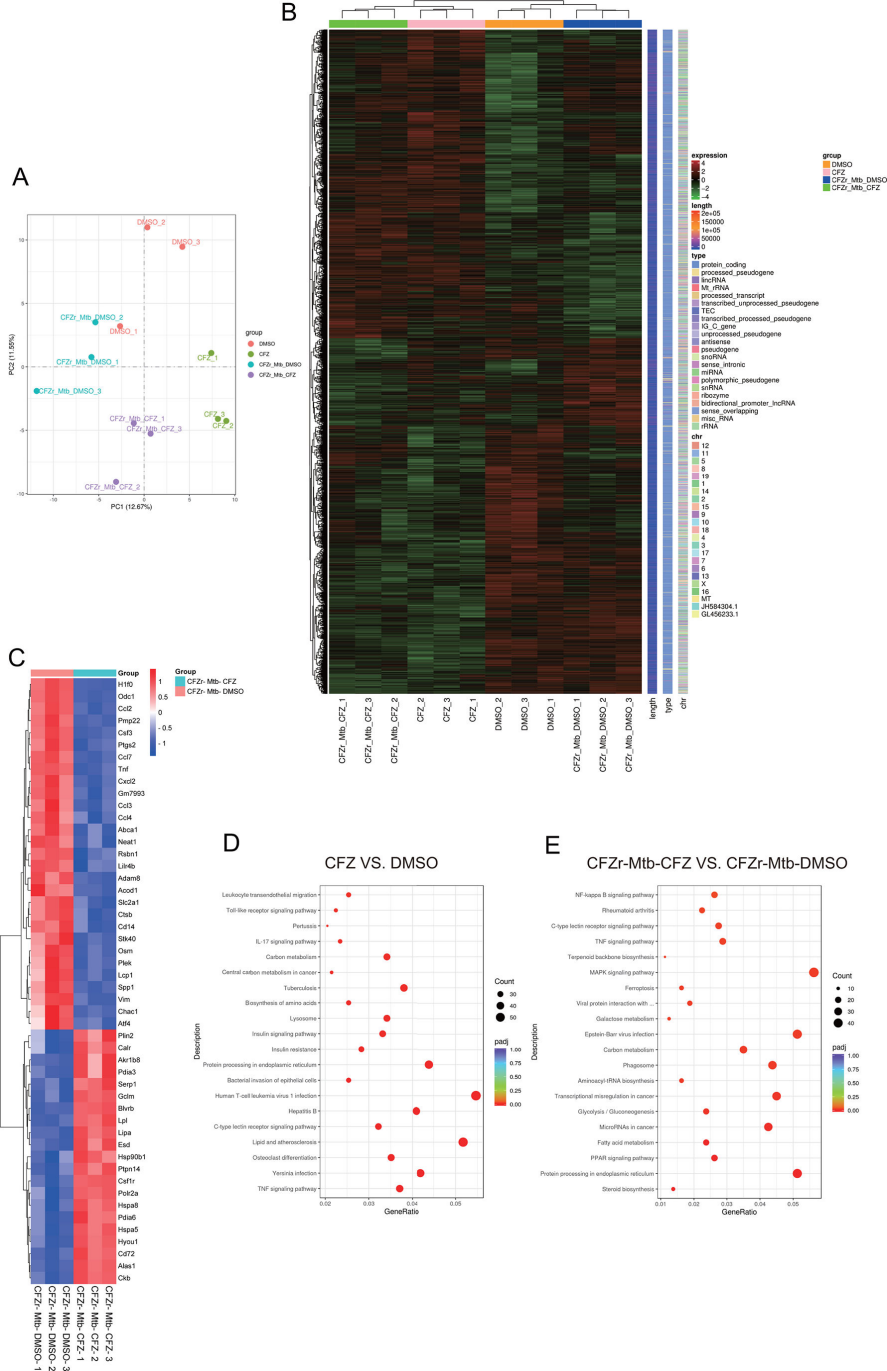
RNA deep sequencing reveals that CFZ inhibits innate immunity. (**A**) PCA between different treatment groups. (**B**) Heatmaps showed differences in gene expression of CFZ treated with 1 µg/mL compared to DMSO with or without CFZr-Mtb infection. (**C**) In the two groups infected with CFZr-Mtb, the expression of the top 50 differentially expressed genes is treated with CFZ or DMSO. (**D and E**) Kyoto Encyclopedia of Genes and Genomes (KEGG) pathway enrichment results of CFZ compared with DMSO group under infection of CFZr-Mtb (**D**) or not (**E**).

The principal component analysis (PCA) plot revealed distinct expression profiles between CFZ vs DMSO and CFZr-Mtb-CFZ vs CFZr-Mtb-DMSO-treated groups (Fig. 5A). Differentially expressed genes (DEGs) with adjusted *P*adj ≤ 0.05 were identified among all group comparisons. A total of 2,064 and 1,589 DEGs were identified in CFZ vs DMSO, CFZr-Mtb-CFZ vs CFZr-Mtb-DMSO, respectively (Fig. 5B). In the CFZr-Mtb infection group, we highlighted the top 50 genes (Fig. 5C), and it was found that many cytokines and chemokines were significantly down-regulated after CFZ treatment, such as colony-stimulating factor 3 (granulocyte) (CSF3), chemokine (C-C motif) ligand 2 (CCL2), chemokine (C-C motif) ligand 7 (CCL7), chemokine (C-C motif) ligand 3 (CCL3), chemokine (C-C motif) ligand 3(CCL4), chemokine (C-X-C motif) ligand 2 (CXCL2), leukocyte immunoglobulin-like receptor, subfamily B, member 4B (LILR4B), the cluster of differentiation 14 (CD14), TNF, and lymphocyte cytosolic protein 1 (LCP1).

We also performed gene set enrichment analysis (GSEA) for Kyoto Encyclopedia of Genes and Genomes (KEGG) pathways and gene ontology (GO) functions to identify molecular pathways associated with host anti-tuberculosis regulation. The results of the non-infected group are shown in Fig. 5D and the infected group are shown in Fig. 5E, which revealed that the immune modification of CFZ may be related to NF-κB and MAPK signaling pathways.

### CFZ negatively regulates the phosphorylation of NF-κB p65

**Fig 6:**
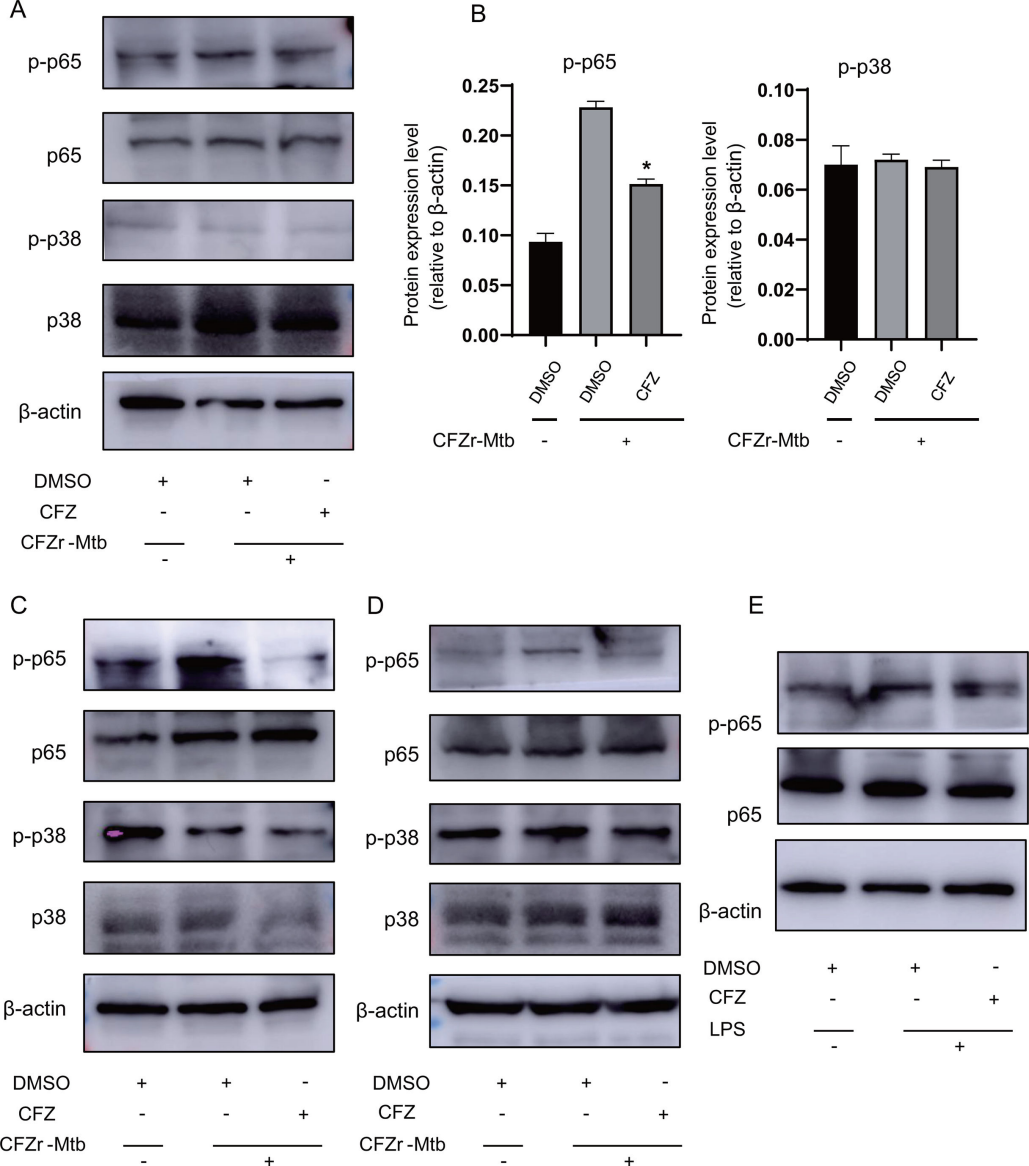
CFZ decreased the phosphorylation of NF-κB p65. (**A, C, and D**) J774A.1 macrophages were infected with or without CFZr-Mtb at an MOI of 0.5 or 1 for 4 h, and treated with CFZ (1 µg/mL) for 12 h or 24 h. The cell lysates were harvested for immunoblotting with antibodies against the indicated proteins. (**B**) Densitometry analysis of the western blots in panel A. (**E**) J774A.1 macrophages were induced with LPS (100 ng/mL) for 12 h and treated with CFZ (1 µg/mL) for 24 h. The cell lysates were harvested for immunoblotting with antibodies against p65, phosphorylated p65, p38, phosphorylated p38 or β-actin.

**Fig 7:**
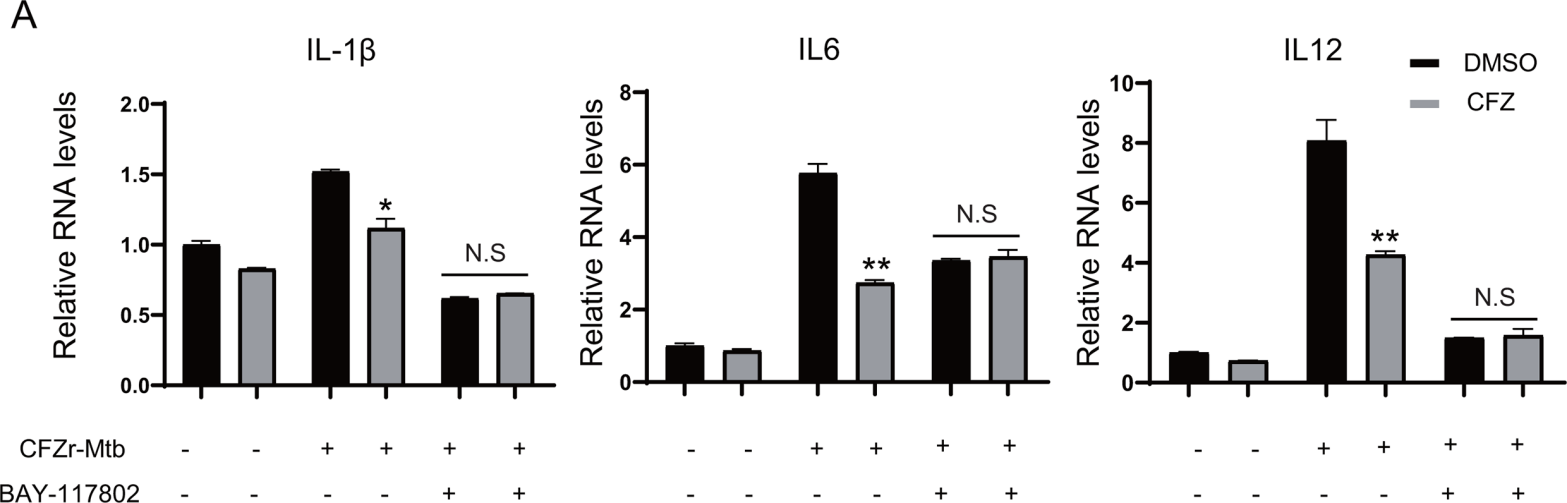
CFZ inhibited innate immunity in a p65-dependent manner. (**A**) J774A.1 macrophages were plated into six wells for 18 h. And then infected with CFZr-Mtb at an MOI of 1 and pretreated with BAY-117052 (10 µM) for 4 h, then continued to be treated with CFZ (1 µg/mL) or DMSO for 24 h. Cells were collected for RNA extraction to detect the transcription level of IL-1β, IL6, and IL12 (*n* = 3; means and SD; *, *P* < 0.05; **, *P* < 0.01; two-sided *t*-test).

Next, to further understand whether the negative regulatory effects of CFZ on innate immunity interfere with NF-κB or MAPK signaling pathways, we infected J774A.1 macrophages with CFZr-Mtb (MOI = 0.5 or MOI = 1) for 4 h and treated them with CFZ for 12 h or 24 h. The cell lysates were harvested for immunoblotting with the indicated antibodies. The data showed that the levels of phosphorylated p65 in CFZ treatment groups were lower than those in DMSO treatment groups (Fig. 6A, B, C, and D). However, phosphorylated p38 was similar. We also examined the effect of CFZ on p65 phosphorylation levels in LPS stimulation models. We treated J774A.1 macrophages with LPS and CFZ for 12 h and detected phosphorylated p65 protein levels. The results were evident with CFZr-Mtb infection (Fig. 6E). To confirm that the effect of CFZ on host innate immunity against Mtb was mainly through NF-kB signaling pathway, we pretreated with BAY-117082, a specific inhibitor of NF-kB, and the effect of CFZ was abolished compared with the control group (Fig. 7A). Overall, the effect of CFZ on innate immunity may be in a p65-dependent manner.

## DISCUSSION

Current TB treatment regimen requires multiple drug combinations and can last up to 6 months for drug-sensitive TB, 18–20 months for MDR/XDR-TB, and possibly longer if new drug resistance is identified (29–34). The failure of the *Mycobacterium tuberculosis* pathogen to clear quickly in a short period of time leads to a sustained attack by the host immune system, which in addition to causing immune dysfunction such as T-cell exhaustion, excessive inflammatory response leads to permanent tissue damage (35–37). TB can lead to irreversible lung damage that appears on radiographic images as scarring, fibrosis, cavitation, or other types of damage (38). This damage can lead to loss of lung function, long-term respiratory symptoms, and eventually chronic respiratory diseases, including chronic obstructive respiratory disease (17). In the current TB treatment regimen, the selection of drug combinations takes culture conversation time as the primary consideration but ignores the immunomodulatory effects of different drugs on the host. Now the host anti-TB immune regulation has been paid more attention, and the concept of host-directed treatment (HDT) of TB has emerged (39, 40). At present, the screening of HDT drugs mainly focuses on the development of new immunomodulatory drugs (41), but there is no reasonable combination and evaluation of the immune effects of drugs in current clinical use. Recently, rifampicin was shown to have immunomodulatory effects especially regulating IL-1β and type I IFN production by modulating macrophage metabolism (42, 43). In this study, we initially evaluated the effects of isoniazid, rifampicin, pyrazinamide, ethambutol, CFZ, TBI-166, and bedaquiline on innate immunity and found that CFZ inhibited cytokines and type I interferons expression, such as IL-6, TNF-α, IL-1β, IFNα, and IFNβ, which provided a theoretical basis for the application of CFZ in alleviating lung tissue injury.

In the course of TB treatment, host immune regulation has opposite dual effects. Innate and adaptive immunity activated by hosts targeting Mtb in the early stage of infection are important protective mechanisms initiated by the host but excessive and prolonged immune activation leads to permanent damage to tissue function. In the later stages of COVID-19 disease, dyspnea may occur and develop into acute respiratory distress syndrome (ARDS) or multiple organ failure (44). Cytokine storms have been reported to be associated with the worsening of many infectious diseases, including severe acute respiratory syndrome (SARS) and Middle East respiratory syndrome (MERS) (45–48). Premature application of this immunosuppressive effect of CFZ may increase susceptibility to other infections. In future drug-resistant TB regimen development, we should consider the CFZ the immunosuppressive effect, begin to use in the appropriate course stage, likely applied to the late period of drug-resistant TB treatment, which may be more beneficial to patient treatment outcomes.

We examined the effect of CFZ on the intracellular survival of CFZ-resistant Mtb, and the results showed that CFZ inhibited the production of inflammatory factors in host cells, but did not promote the growth of CFZr-Mtb. Type I interferons facilitate Mtb parasitism in the host, and Liu et al. found that IFNβ induces the formation of lipid droplets that facilitate the intracellular survival of *M. tuberculosis* (49). We proposed that the Mtb inhibition effect of IL-6, TNF-α, and IL-1β and the Mtb promotion effect of IFNα and IFNβ cancel each other out. However, cytokines and chemokines are important components that affect the activation of adaptive immunity, and CFZ’s effect on innate immunity may inhibit the establishment of specific immunity. Therefore, we speculate that CFZ may not be suitable for immediate application in the early stage of disease infection or in immunocompromised populations such as AIDS patients. We will also analyze the different treatment outcomes of CFZ in patients with different immune status in the next step. In summary, CFZ may have a more prominent effect in the later stage of TB treatment, combining anti-inflammatory and antibacterial prominent effects.

## MATERIALS AND METHODS

### Cell lines and mycobacterial strains

HEK293T (ATCC CRL-3216, RRID: CVCL_0063), J774A.1 (ATCC TIB-67, RRID: CVCL_0358), and THP-1 (ATCC TIB-202, RRID: CVCL_0006) were obtained from the American Type Culture Collection (ATCC). HEK293T cells and J774A.1 cells were maintained in Dulbecco’s modified Eagle’s medium (Gibco, USA) supplemented with 10% fetal bovine serum (FBS) (Gibco, USA). THP-1 cells were cultured in RPMI 1640 medium supplemented with 10% FBS. All cells were cultured at 37°C in 5% CO_2_. *M. tuberculosis* H37Rv (ATCC 27294). Clinical CFZ-resistant strain of *M. tuberculosis* (CFZr-Mtb) for this study was obtained from the National Clinical Laboratory on Tuberculosis in Beijing Chest Hospital. *M. tuberculosis* strain H37Rv and clinical isolates were cultured in Middlebrook 7H9 broth with 0.2% (vol/vol) glycerol, 0.05% Tween-80, and 10% (vol/vol) oleic acid-albumin-dextrose-catalase (OADC).

### Drugs, plasmids and antibodies

Clofazimine was purchased from Sigma-Aldrich; TBI-166 was provided by the Institute of Materia Medica, Peking Union Medical College, and Chinese Academy of Medical Sciences; lipopolysaccharides from *Escherichia coli* O55:B5 (L2880) was purchased from Sigma-Aldrich. BAY11-7082 (M2040), SP600125 (M2076), and U0126 (M1977) were purchased from Abmole.

Luciferase reporter assay plasmids for pRL-TK, Gal4-Elk, Gal4-Luc, pFA-cJun, RacL61, pNF-κB-luc, and RasV12 were kindly provided by Cuihua Liu (Institute of Microbiology, Chinese Academy of Sciences).

The following antibodies were used in this study: anti-p-Jnk (sc-81502, Santa Cruz, USA), anti-Jnk (9252, CST, USA), anti-p-p38 (sc-17852-R, Santa Cruz, USA), anti-p38 (sc-7972, Santa Cruz, USA), anti-p-IκBα (sc-101713, Santa Cruz, USA), anti-IκBα (sc-371; Santa Cruz, USA), p-ERK1/2 (9101, CST, USA), and ERK1/2 (9102, CST, USA).

### Luciferase reporter assays

NF-KB, AP-1, and ELK promoter activation were detected with the Promega luciferase reporting assay kit. HEK293T cells were inoculated in 24-well plates for 12 h and transfected with the corresponding plasmid. For the detection of NF-κB, pNF-κB-Luc (0.5 µg), and pRL-TK (25 ng) were transfected, replaced by 10% FBS DMEM medium with CFZ or DMSO after 6 h, and TNF-α (20 ng/mL) was added after 18 h for 6 h. For AP-1 detection, cells were transfected with Gal4-luc (0.45 µg), pFA-cJun (0.15 µg), RacL61 (0.25 µg), and pRL-TK (25 ng) for 6 h and then replaced with medium containing CFZ or DMSO. For Elk detection, cells were transfected with Gal4-luc (0.3 µg), Gal4-Elk (0.3 µg), pRL-TK (25 ng), and RasV12 (5 ng).

### Quantitative RT-PCR analysis

Total RNA was isolated from cells using TRIzol (Invitrogen, USA). Then, cDNA was generated from the total RNA using cDNA Synthesis SuperMix (Yeasen, China). Quantitative RT-PCR (RT-qPCR) was performed using SYBR Green Real-time PCR Master Mix (Yeasen, China) with specific primers. The following primers were used: IL6-forward: TACCACTCCCAACAGACC reverse: CATTTCCACGATT TCCCAGA; IL1β-forward: GCCACCTTTTGACAGTGATG reverse: TGATGTGC TGCTGCGAGA; TNFα-forward: TCTCATTCCTGCTTGTGG reverse: ACTTGGT GGTTTGCTACGA; GAPDH-forward: CAAATTCAACGGCACAGTCA reverse: T TAGTGGGGTCTCGCTCC; IFNα-forward: CCTGTGTGATGCAGGAACC reverse: TCACCTCCCAGGCACAGA IFNβ-forward: ACTAGAGGAAAAGCAAGAGGA reverse: CTGGTAAGTCTTCGAATGATG; IL12-forward: CTGTGCCTTGGTAG CATCTATG reverse: GCAGAGTCTCGCCATTATGATTC.

### Western blotting

The cell culture supernatants were removed, washed with PBS, and proteins were extracted by RIPA lysis buffer (R0010, Solarbio, China) with protease inhibitors (P1010, Beyotime, China) and phosphatase inhibitors (P1051, Beyotime, China). The cell lysis mixture was transferred into an EP tube, centrifuged (4°C, 12,000 *× g*, 10 min), and the supernatants were collected. The separation gel with appropriate concentration was prepared according to the molecular weight of the target protein. The electrophoresis conditions were 80 v 30 min and then 120V until bromophenol blue ran out of the separating gel. Semi-dry transfer conditions: 15 v, 1 h (time adjusted according to protein molecular weight). The membranes were blocked with 5% skim milk or 5% BSA at room temperature for 1 h. After TBST (10 mM Tris, 150 mM NaCl, 0.05% Tween-20, pH 7.2–7.6) washing for four times, the primary antibodies were incubated overnight at 4°C. Remove the primary antibody, wash with TBST for four times, and incubate with the horseradish peroxidase-conjugated secondary antibody at room temperature for 1 h. After washing for four times, add color-developing solution (P0018, Beyotime, China) for exposure (Amersham Imager 600).

### ELISA

Macrophage culture supernatant was used to detect IL-6 levels after CFZ treatment under LPS treatment. THP-1 cells induced into adherent macrophages by 100 ng/mL PMA were prepared into a final concentration of 1 × 10^6^cells/mL. The cells were treated with CFZ for 24 h and induced by LPS for 12 h. The collection was transferred to an EP tube, centrifuged in 10 min to collect the supernatant, and the IL-6 concentration was detected by ELISA kit (SEKH-0013, Solarbio, China) according to the manufacturer’s instructions. Briefly, 100 µL of the samples was added to the corresponding culture plate hole and incubated at 37°C for 90 min, and then the liquids were removed. Immediately 100 µL biotinylation antibody working solution was added, incubated at 37°C for 60 min, and removed. After washing three times, 100 µL HRP enzyme conjugate working solution was added, incubated at 37°C for 30 min, washed three times, 90 µL TMB thick was added, and incubated at 37°C for about 15 min. 50 µL of Stop solution was added to each well. Immediately read and process the data at 450 nm wavelength. The plate reader used was Varioskan LUX (ThermoFisher, USA).

### Intracellular activity assay

J774A.1cells were digested with trypsin and prepared into a final concentration of 4 × 10^5^ cells/ml, and infected with *M. tuberculosis* strains H37Rv at indicated MOI for 4 h. After washing three times with prewarmed sterile phosphate-buffered saline (PBS) to remove extracellular bacteria. The tested drugs were added to the medium and the J774A.1 macrophages were re-incubated for another 48 h. The infected macrophages were washed with PBS to remove compound-containing medium, and then the macrophage cells were lysed with SDS lysis buffer (Sigma-Aldrich). The original culture and 10-fold serial dilutions of culture suspension were extracted and plated on Middlebrook 7H10 agar supplemented with 10% (vol/vol) OADC and 0.2% (vol/vol) glycerol for 3–4  weeks.

### Statistical analysis

All data were presented as the mean ± standard deviation. Statistical analysis was performed using GraphPad Prism 8 software (GraphPad Software, Inc.). The experimental group means were compared to the untreated group by two-sided *t*-test. *P* value of 0.05 was considered significant.

## SUPPLEMENTAL MATERIAL

Figure S1 msphere.00254-24-s0001.eps Effects of currently commonly used anti-tuberculosis drugs on host innate immune signaling pathways.

Supplemental caption msphere.00254-24-s0002.docx Caption for Fig. S1.

ASM does not own the copyrights to Supplemental Material that may be linked to, or accessed through, an article. The authors have granted ASM a non-exclusive, world-wide license to publish the Supplemental Material files. Please contact the corresponding author directly for reuse.
