# Supplementary material for: Clofazimine inhibits innate immunity against Mycobacterium tuberculosis by NF-κB
Source: mSphere. 2024 Jul 24;9(8):e00254-24. doi: 10.1128/msphere.00254-24 (PMC11351037; doi:10.1128/msphere.00254-24)
Supplement: Supplemental caption — Caption for Fig. S1. [file msphere.00254-24-s0002.docx]

**Supplementary Information for: Clofazimine inhibits innate immunity** **against Mycobacterium tuberculosis by NF-****κB**

Xinda Li, ^1,2^^,3^ Xiaoyi Luo, ^1,2,3^ Bin Wang, ^1,2^ Lei Fu, ^1,2^ Xi Chen, ^1,2^ Yu Lu, ^1,2,4,^ *

^1^ Department of Pharmacology, Beijing Chest Hospital, Capital Medical University, Beijing, China

^2^ Beijing Key Laboratory of Drug Resistance Tuberculosis Research, Beijing Tuberculosis and Thoracic Tumor Research Institute, Beijing, China

^3^These authors contributed equally

^4^Lead contact

*Correspondence: luyu4876@hotmail.com (Y.L.)

Contents:

Figure S1. Effects of currently commonly used anti-tuberculosis drugs on host innate immune signaling pathways.

(A) HEK293T cells were seeded into 24 wells, transfected with NF-κB, AP-1, or Elk luciferase reporter plasmid and treated with isoniazid (INH, 5μg/ml)), rifampicin (RFP, 5μg/ml), pyrazinamide (PZA, 5μg/ml), ethambutol (EMB, 5μg/ml), bedaquiline (BDQ, 5μg/ml) or an equal volume of DMSO for 24h.The Elk was activated by co-expression of constitutively active RasV12. The AP-1 was activated by co-expression of constitutively active RacL61. The NF-κB pathway was stimulated by TNF treatment. BAY-117802, a known NF-κB inhibitor, SP600125, a known AP-1 inhibitor, and U0126, a known ERK inhibitor, were used as positive controls in this study. Then, the cell lysates were harvested for a luciferase assay. (n = 3; means and SD; *, p < 0.05; **, p<0.01; two-sided *t*-test).
